# IRAK3 is upregulated in rheumatoid arthritis synovium and delays the onset of experimental arthritis

**DOI:** 10.3389/fimmu.2025.1468341

**Published:** 2025-04-30

**Authors:** Federica Borghese, Richard O. Williams, Felix I. L. Clanchy

**Affiliations:** ^1^ Kennedy Institute of Rheumatology, University of Oxford, Oxford, United Kingdom; ^2^ Botnar Institute for Musculoskeletal Sciences, Nuffield Department of Orthopaedics, Rheumatology and Musculoskeletal Sciences, University of Oxford, Oxford, United Kingdom

**Keywords:** arthritis, inflammation, IRAK3, macrophages, cytokines

## Abstract

Tumour necrosis factor (TNF) is a potent inducer of endotoxin tolerance-associated molecules, such as interleukin-1 receptor-associated kinase 3 (IRAK3), and also a therapeutic target in inflammatory autoimmune diseases, as it upregulates the production of inflammatory mediators. The role of IRAK3 was assessed in rheumatoid arthritis (RA), a disease which is amenable to TNF blockade. As a variant of IRAK3 lacks the death domain required for its canonical role, isoform expression was determined in different inflammatory milieu by immunoblotting. RA synovial explant expression of IRAK3 was measured by qPCR. The expression of the larger, “classical” IRAK3 isoform predominated in macrophages treated with various stimuli. The expression of IRAK3 was higher in RA synovium compared to osteoarthritis synovium. Using collagen-induced arthritis, a murine model of RA, the immunomodulatory role of IRAK3 was investigated with wild-type (WT) and IRAK3-deficient mice expressing the MHC-II A^q^ allele. Disease progression was significantly accelerated in IRAK3^−/−^ mice. In addition, the circulating levels of IL-1β were greater, and there were fewer Tregs both before and after the onset of disease. Inflammatory gene expression was higher in the arthritic paws of IRAK3^−/−^ mice. This study demonstrates that IRAK3 deficiency accelerates the progression of arthritis and increases molecular markers of disease severity.”

## Introduction

Rheumatoid arthritis (RA) is a chronic inflammatory disease that primarily affects the joints and, without adequate treatment, results in progressive destruction. It is more frequent in women than men, and its prevalence is 0.5%–1% in developed countries (1). Although the cause of RA is not fully elucidated, genetic and environmental factors are associated with susceptibility to the disease.

The efficacy of tumour necrosis factor (TNF) blockade in combination with methotrexate for the treatment of RA indicates a central role for TNF in disease progression. However, TNF has both pro-inflammatory and immunomodulatory actions, and several studies have demonstrated that TNF blockade can prevent endotoxin tolerance (ET), due in part to a reduction in interleukin-1 receptor-associated kinase 3 (IRAK3) expression (2). IRAK3 (also known as IRAK-M) is a member of the IRAK family, together with IRAK1, 2 and 4, and is expressed predominantly in macrophage (Mφ)-lineage cells (3). By preventing signaling via MyD88, IRAK3 is a pivotal inhibitor of the Toll-like receptor (TLR) and IL-1β/IL-18 signaling pathways. The IRAK proteins have several structural similarities such as a death domain, kinase/pseudo-kinase domain, and, excepting IRAK4, a TRAF6 binding domain (4, 5). As a common denominator of the IRAK proteins, the death domain facilitates intermolecular binding between IRAKs (6). Lacking kinase activity, IRAK3 inhibits the MyD88 pathway by associating with IRAK4 via their respective death domains, thereby altering the normal assembly of the MyD88–IRAK4–IRAK1 signaling complex (4). Finer mapping of the functional structures of IRAK3 has revealed putative key amino acids necessary for its activity (7, 8). An isoform of IRAK3 lacks most of the death domain, and its function is largely unknown, but mutations and deletions in this region can reduce the inhibition of inflammatory mediator secretion in Mφ-lineage cells, suggesting that it is unable to perform the canonical inhibitory function of the classical variant (6).

Several studies have indicated a role for IRAK3 in reducing or delaying inflammatory disease. For example, the accelerated development of asthma is associated with variants of *IRAK3* in multiple studies, the first of which identified several potentially pathogenic mutations in the protein-coding region (9–11); *IRAK3* is also a key gene biomarker in the peripheral blood of childhood asthma patients with exacerbation of disease activity (12). IRAK3 suppresses a murine model of systemic lupus erythematosus (SLE) (13), but a trend towards the association of genetic variants of IRAK3 was not statistically significant in a study of a European SLE population (14). A deficiency of IRAK3 in an atherosclerosis murine model led to an exacerbation of disease activity (15). IRAK3 is one of eight core genes associated with osteoarthritis (OA) in patients with metabolic syndrome (16). In circulating monocytes, the expression of IRAK3 was higher in RA patients with low/moderate disease activity compared to high disease activity (17). In RA fibroblast-like synoviocytes, the expression of IRAK3 (and other IRAK family members) is increased by TLR ligands (18). IRAK3 can also contribute to the development of disease. IRAK3 is upregulated in IgG4-related disease manifesting in salivary gland inflammation with M2 Mφ-associated fibrosis (19); fibrosis was also promoted by IRAK3 in a bleomycin-induced lung injury model (20). IRAK3 has been demonstrated to promote some forms of cancer (21–23).

The cytokines IL-1β and TNF are highly expressed within the inflamed RA joint and, by binding to their respective receptors, cause inflammatory signaling, including the NF-κB signaling pathway (24, 25). Inflammation-driven tissue remodeling also releases Damage-associated molecular patterns (DAMPs), which triggers TLR signaling (26). In the OA joint, inflammation is driven by the IL-1β and mechano-sensitive signaling pathways rather than the significant leucocyte infiltrate and synovial hyperplasia observed in RA (27). TNF induces the expression of regulatory signaling proteins such as IRAK3; in time, IRAK3 attenuates signaling via TLR/IL-1β, including the NF-κB pathway.

This study’s purpose was to investigate IRAK3-associated mechanisms in the context of a chronic inflammatory disease for which anti-TNF is efficacious (28), as its expression has been shown to be dependent on TNF *in vitro* (2). The expression of IRAK3 was determined in human Mφ given a range of stimuli and in human RA synovial explants. IRAK3 has two main isoforms, and it was established that Mφ predominantly express the longer, classical isoform of IRAK3 in Mφ given a range of stimuli; IRAK3 expression was higher in RA synovium compared to OA synovium. To investigate the systemic effects of IRAK3, collagen-induced arthritis (CIA) was induced in mice lacking this gene. The development of arthritis was accelerated and characterized by increased circulating levels of IL-1β and reduced IL-5. While regulatory T cells (Tregs) were reduced in the lymph nodes (LNs) of IRAK3^−/−^ during CIA, there was an increased T-cell gene expression profile in IRAK3^−/−^ arthritic joints, compared to wild-type (WT) joints.

## Materials and methods

### Irak3 KO mice

Mice lacking IRAK3 (B6.129S1-IRAK3^tmlFlv^/J) with a C57BL/6 background, were kindly provided by Hans-Joachim Anders courtesy of Koichi Kobayashi and Richard Flavell; the genetic modification introduces a stop mutation resulting in a significantly truncated mutant transcript (29). Knockout (KO) status was screened using the following primers: forward primer 5′-CGTTCCATAACACACCTCTCTGC-3′; reverse primer 5′-TTCTATCGCCTTCTTGACGAGTTC-3′. WT status was determined using the following primers: forward primer 5′-GCCAGAAGAATACATCAGACAGGG-3′; reverse primer 5′-TGTTTCGGGTCATCCAGCAC-3′. C57BL/6 IRAK3^−/−^ mice (which express the MHC-II allele A^b^) were crossed with the congenic C57BL/6NQ mice (expressing the MHC-II allele A^q^) to increase susceptibility to arthritis (30); C57BL/6NQ mice were generously provided by Johan Bäcklund (Karolinska Institute). Mice were crossed to create heterogeneous breeding pairs, which were further crossed and their progeny screened for H2^q^ homozygotic mice that were homozygous for a lack of *Irak3*; see **Supplementary Table 1** for primers. All mice were housed in pathogen-free conditions with *ad libitum* food and water at the Kennedy Institute of Rheumatology (KIR). All procedures were conducted in accordance with UK Home Office regulations and guidelines.

### Collagen-induced arthritis

Male C57BL/6NQ IRAK3^−/−^ and C57BL/6NQ WT mice were immunized with bovine type II collagen in complete Freud’s adjuvant as previously described (31). After mice developed clinical arthritis, the disease severity in the paws was scored as follows: 0, normal; 1, slight swelling and/or erythema; 2, clear swelling; and 3, pronounced edematous swelling/ankylosis. Clinical parameters were measured for 10 days after disease onset in each animal, and then animals were euthanized; blood plasma, spleens, inguinal lymph nodes, and paws were harvested. For pre-onset studies, tissue harvests took place 14 days after immunization.

### Phenotyping cell subsets by flow cytometry

To analyze Th lymphocyte subsets, cells from the spleen and LN were isolated as previously described (28). Leucocytes were directly stained with viability dye (Zombie NIR Fixable Viability dye, BioLegend, San Diego, CA, USA), and antibodies for CD4 (RM4-5, BioLegend) and CD25 (PC61, BioLegend); then fix/permeabilized and stained intra-cellularly for FoxP3 (236A/E7, eBioscience, San Diego, CA, USA), Gata3 (TWAJ, eBioscience), RORγt (Q31-378, BD Biosciences, San Jose, CA, USA), Tbet (4BD10, BioLegend), and Helios (22F6, BioLegend); and analyzed using the FACSDIVA software (BD Biosciences) and FlowJo (TreeStar). To quantify T-cell subsets, live cells were gated into Tregs (CD4^+^CD25^+^FoxP3^+^), natural Tregs (CD4^+^CD25^+^FoxP3^+^Helios^+^), Th2 (CD4^+^Gata^+^), Th1 (CD4^+^Tbet^+^), and Th17 (CD4^+^RORγt^+^) cells.

### Murine plasma biomarkers

Blood was collected via cardiac puncture into microcentrifuge tubes containing heparin. Samples were then centrifuged at 13,000 rpm for 10 minutes at 4°C. Plasma was then stored at −80°C until the measurement of anti-collagen responses as previously described (32). Inflammatory mediators in murine plasma were measured using the Mouse TH1/TH2 9-Plex Ultra-Sensitive Kit (MesoScale Discovery, Rockville, MD, USA; catalogue number K15013C-1) according to the manufacturer’s instructions.

### Gene expression in murine tissue

Paws were snap-frozen in liquid nitrogen and processed as previously described (31). Briefly, tissue was pulverized with the BioPulverizer™ (BioSpec, Bartlesville, OK, USA). Paw powder was then homogenized in 500 μL of TRIzol reagent (Invitrogen, Carlsbad, CA, USA) using the Sample Grinding Kit (GE Healthcare, Chicago, IL, USA). Chloroform (100 μL) was added to the tube, and the lysate was mixed and then centrifuged to separate the mixture into a lower phenol phase and upper aqueous separated by an interphase. The aqueous phase of the phenol/chloroform extraction was mixed with an equal volume of ethanol and then added to an RNA extraction column (miRNeasy Mini Kit, Qiagen, Valencia, CA, USA), and mRNA extraction was completed according to the manufacturer’s instructions.

cDNA was reverse transcribed from 500 ng of RNA using a High Capacity cDNA Reverse Transcription Kit (Applied Biosystems, Foster City, CA, USA), according to the manufacturer’s protocol. The expression of target genes was determined using TaqMan assays (Thermo Fisher Scientific, Waltham, MA, USA) and qPCR master mix (Takyon Low ROX Probe 2x dTTP blue, Eurogentec, Seraing, Belgium) and was expressed relative to *Gapdh* gene expression using the ΔΔCT approximation method; see **Supplementary Table 2** for TaqMan assays. Murine Mφ from mice lacking either *Tnfrsf1a* or *Tnfrsf1b* were derived from bone marrow as previously described (33) and stimulated for up to 12 hours with Lipopolysaccharide (LPS) (10 ng/mL) before being processed for RNA extraction, reverse transcription, and qPCR.

### Human IRAK3 isoforms

“Classical” full-length IRAK3 (producing NP_009130.2) and “alternative” death domain-truncated IRAK3 (producing NP_001135995.1) were cloned by traditional molecular biology techniques and transfected into HEK293 cells using Lipofectamine 2000 (Thermo Fisher Scientific). After 24 hours, cells were lysed for extraction using the Proteome Profiler Human NFkB Pathway Array kit (R&D Systems, Minneapolis, MN, USA). Human monocyte-derived Mφ (MDM) were derived from monocytes and stimulated as previously described (33). Briefly, MDM were differentiated from monocytes for 5 days in 10% foetal bovine serum supplemented Roswell Park Memorial Institute 1640 medium (FBS RPMI) (10^7^ cells/10 mL/10-cm dish) supplemented with 50 ng/mL macrophage-colony stimulating factor (M-CSF) (PeproTech, Cranbury, NJ, USA); MDM were re-plated into 12-well plates, rested overnight, and then stimulated with medium alone, LPS (10 ng/mL, Merck, Darmstadt, Germany), LPS + IFNγ (10 ng/mL + 10 ng/mL, Merck/PeproTech), TNF (50 ng/mL, PeproTech), granulocyte macrophage-colony stimulating factor (GM-CSF) (50 ng/mL, PeproTech), IL-4 (50 ng/mL, PeproTech), or TGF-β1 (100 ng/mL, PeproTech) for 20 hours; protein and RNA were extracted using an Isolate II RNA/DNA/Protein kit (Bioline, Luckenwalde, Germany). Protein concentration was determined (Pierce™ BCA Protein Assay Kit, Thermo Fisher Scientific), and 4 µg of protein was boiled in Laemmli buffer (Alfa Aesar), run on a polyacrylamide gel (NuPAGE™ 4%–12% Bis-Tris Mini Protein Gels, Thermo Fisher Scientific), and then transferred to a nitrocellulose membrane for immunoblotting. IRAK3 was detected using the antibody clone SAB3500193 (Merck) or AF6264 (R&D Systems) according to the manufacturer’s instructions; β-actin was detected using AC-74 (Merck).

Synovial explants from RA and OA patients were processed as previously described (34). Briefly, synovial membrane tissue was obtained from RA and OA patients’ joints, dissected from the surrounding tissues, and digested *in vitro* with Liberase TL (Merck) and DNAseI (Merck) diluted in RPMI. After incubation at 37°C for 1–2 hours, the digestion was halted by the addition of RPMI containing 10% FBS, and the digestion passed through a cell strainer and washed, and erythrocyte lysis was performed. The cells were then washed and pelleted for RNA extraction and reverse transcription. Gene expression was measured by standard curve qPCR using a linearized plasmid containing the “classical” *IRAK3* isoform and housekeeping gene (*HPRT1*); tissues were collected in compliance with approval from the Riverside Research Ethics Committee. The gene expression of *IRAK3* in MDM was determined by ΔΔCT approximation.

### Statistical analyses

Data were analyzed using Excel (Microsoft Ltd.), Prism (GraphPad Software Ltd.), and MultiExperiment Viewer (TM4 software). Student’s t-test or one-way ANOVA with *post-hoc* analysis was used to test statistical significance. A p-value <0.05 was considered statistically significant.

## Results

### The larger, “classical” IRAK3 isoform predominates in Mφ

**Figure 1:**
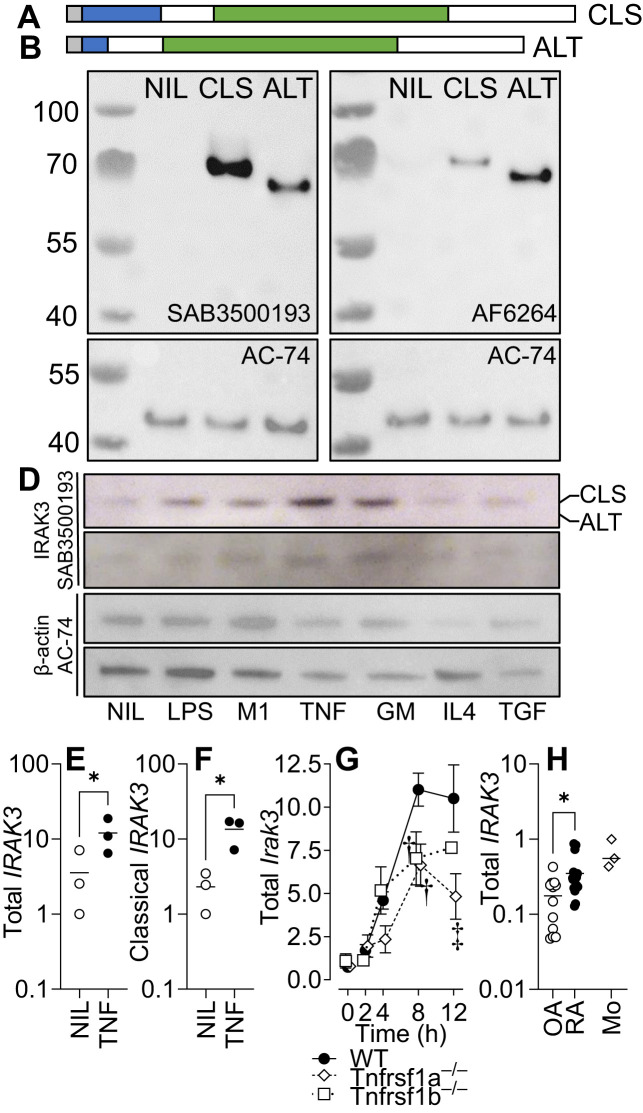
IRAK3 isoform expression in primed macrophages. IRAK3 N-terminal to death domain (DD) (a.a. 1–14) is shaded grey, DD (15–104) is shaded blue, and pseudokinase domain (a.a. 171–446) is shaded green. **(A)** “Classical” full-length (CLS; NP_009130.2 a.a. 1–596). **(B)** “Alternative” IRAK-3 (ALT; NP_009130.2 a.a. 1–44, 105–596 of CLS) that lacks most of the DD. **(C)** Western blotting for IRAK3 in HEK293 cells (NIL) or HEK293 cells overexpressing classical or alternative IRAK3 blotted with two antibodies for IRAK3. **(D)** IRAK3 expression in monocyte-derived Mφ, unstimulated (NIL) or stimulated for 20 h with LPS, LPS/IFNγ (M1), TNF, GM-CSF (GM), IL-4, or TGF-β1 (TGF); representative blots of two donors stained for IRAK3 (SAB3500193) and β-actin (AC-74). Gene expression of **(E)** both *IRAK3* isoforms and **(F)** the classical *IRAK3* isoform in TNF-stimulated human Mφ. **(G)** Gene expression of *Irak3* in LPS-stimulated (10 ng/mL) murine bone marrow-derived Mφ from wild-type or transgenic mice lacking either *Tnfrsf1a* or *Tnfrsf1b*; ANOVA for each respective knockout (KO) vs. wild type (WT), † p < 0.01, ‡ p < 0.001, n = 3–5 experiments. **(H)** Total *IRAK3* gene expression in osteoarthritis (OA) and rheumatoid arthritis (RA) synovial explants and normal healthy monocytes (Mo), n = 12 OA, 19 RA, and 3 Mo. For **(E, F, H)** * p < 0.05, Student’s t-test.

We and others have demonstrated the differential expression of splice variants with different functional properties in inflammatory conditions (33, 35). Although most research has focused on the larger variant of IRAK3, which has the full-length death domain necessary for associating with the Myddosome complex (6), we demonstrated that two commercially available antibodies were able to detect both isoforms (**Figures 1A–C**) before determining whether mature Mφ primed with different inflammatory stimuli (including TNF) expressed different proportions of each isoform. Only the larger isoform was observed in human MDM primed with different stimuli (**Figure 1D**, see **Supplementary Figure 1** for quantification). As previously observed (2), the expression of IRAK3 is induced by TNF (**Figures 1E, F**); however, murine Mφ from Tnfrsf1^−/−^ mice had significantly reduced the expression of IRAK3 compared to WT or Tnfrsf2^−/−^ mice (**Figure 1G**). In synovial explants from OA and RA patients, the expression of IRAK3 was measured by standard curve qPCR; IRAK3 was found to be higher in RA patient samples (**Figure 1H**).

### Creation of an IRAK3^−/−^ mouse susceptible to CIA

**Figure 2:**
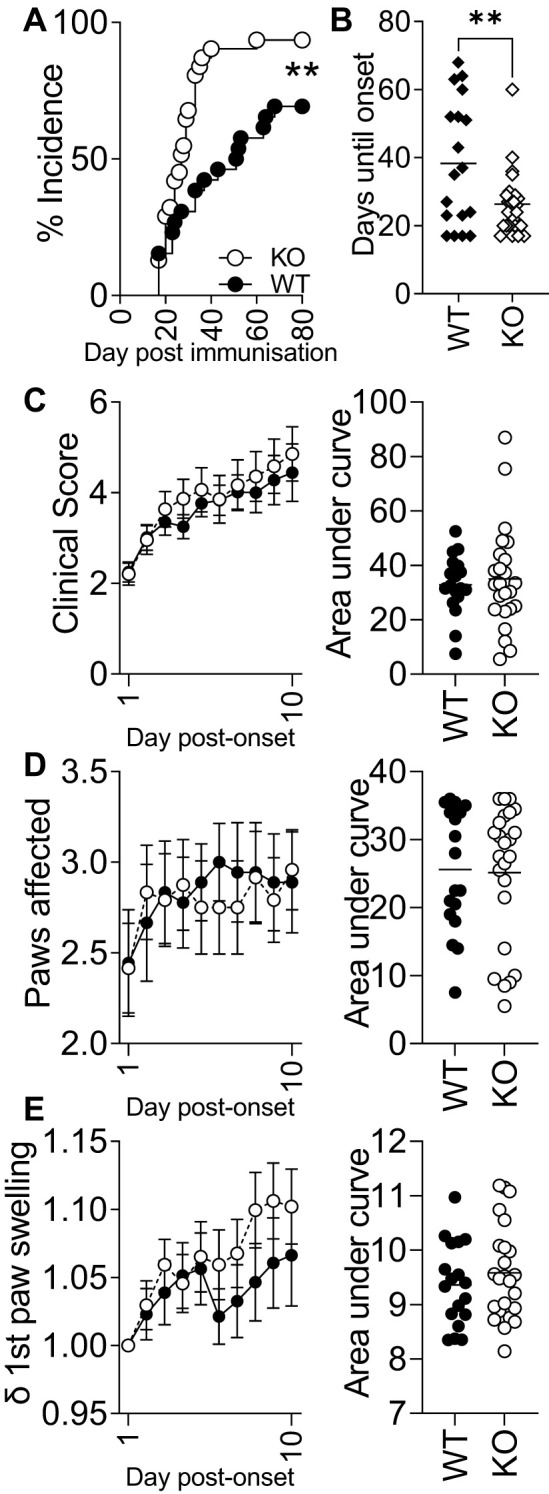
Experimental arthritis onset is accelerated and more efficient in IRAK3^−/−^ mice. **(A)** IRAK3^−/−^ mice [Knockout (KO)] develop arthritis significantly earlier than wild type (WT) (Mantel–Cox test), taking on average half the time to develop symptoms **(B)**; ** p < 0.01, Student’s t-test. **(C)** Global clinical score, **(D)** the paws affected for each mouse, and **(E)** change in swelling in the individual first affected paw relative to day 0 were assessed daily during the post-onset 10-day observational period. Data are combined from two independent experiments with a total of 18 (WT) and 24 (KO) mice per group; values are mean ± SEM.

**Figure 3:**
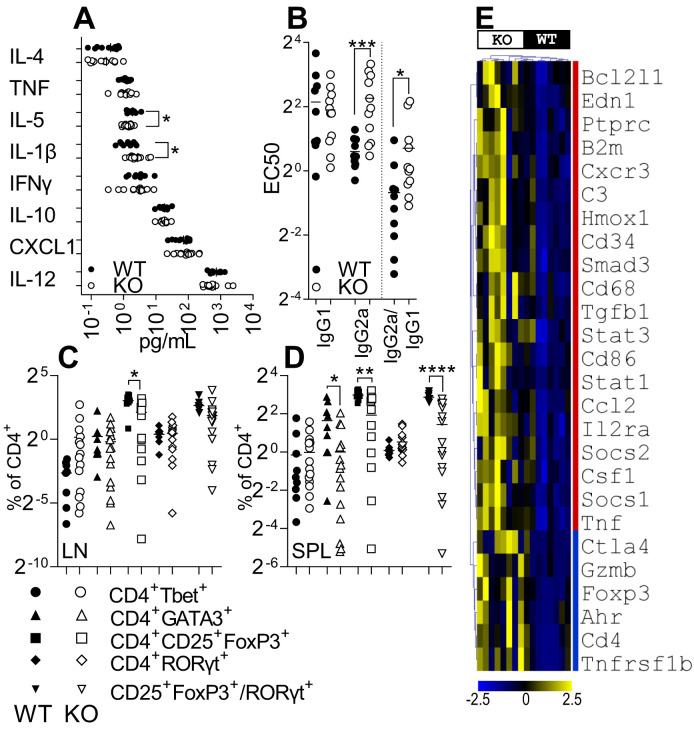
IRAK3 deficiency exacerbates inflammatory mediator expression in blood and paws. **(A)** Cytokines measured in plasma from arthritic IRAK3^−/−^ (KO) and wild-type (WT) mice; n = 10 and 14, respectively. **(B)** EC_50_ of anti-collagen IgG1 and IgG2a in the plasma. Live CD4^+^ subsets measured in **(C)** lymph nodes (LN) and **(D)** spleens (SPL) of arthritic mice at day 10 post-onset for WT vs. KO. **(E)** Statistically significantly different inflammatory gene expression in affected paws of mice at day 10 post-disease onset; see **Supplementary Figure 5** for all 91 genes measured, n = 8 per group. For **(A–E)**, *p < 0.05, **p < 0.01, ***p < 0.001, and ****p < 0.0001.

As IRAK3 variants have an association with accelerated and exacerbated disease activity in humans, we bred a transgenic Irak3^−/−^ mouse onto a background suitable for CIA in order to determine how the lack of this gene altered experimental disease. The murine MHC-II A^q^ molecule has a high degree of similarity to HLA-DRB1*0101 and *0401, which confer susceptibility to human RA (30). Most genetically modified mouse strains have a C57BL/6 (H2^b^) background, which is resistant to the development of autoreactive T-cell responses to type II collagen in CIA. Hence, it has been proposed that C57BL/6NQ mice (expressing A^q^) serve as an international standard in studies of T cell-driven immunopathology (30). We crossed IRAK3^−/−^ mice onto C57BL/6NQ mice after developing a PCR method for genotyping H2^b^ and H2^q^ mice; this necessitated the sequencing of relevant genomic regions of each strain (**Supplementary Figure 2A**) and a comparison of genotyping by flow cytometry and PCR (**Supplementary Figures 2B–D**, **3**). The IRAK3^−/−^ NQ mice were used for CIA, with disease induction being performed as previously described (33). As shown in **Figure 2A**, the progression of the disease in IRAK3^−/−^ NQ mice was significantly more rapid, with the average time to arthritis onset being 26 versus 38 days in the WT group (**Figures 2A, B**). Disease activity was similar, although there was a trend towards increased severity in IRAK3^−/−^ mice, as indicated by greater paw swelling at later time-points (**Figures 2C–E**); this was consistent with significantly higher levels of IL-1β and lower levels of IL-5 detected in the plasma of IRAK3^−/−^ NQ mice (**Figure 3A**).

### Accelerated arthritis in IRAK3^−/−^ mice is associated with reduced peripheral Tregs

To further investigate the differences in kinetics and severity of disease between WT and KO mice, an analysis of auto-antibodies and Th lymphocyte subsets was performed to assess systemic changes between strains. Anti-collagen auto-antibodies were measured in the plasma of arthritic animals to determine humoral responses and infer cytokines present in proximity to auto-reactive B cells; IgG1 and IgG2a anti-collagen antibodies were measured to determine class switching. Surprisingly, while the level of IgG1 antibodies was virtually the same in both strains, the EC_50_ for a serial dilution analysis was higher for IgG2a antibodies in the IRAK3^−/−^ group, indicating lower titers for this Ig class (**Figure 3B**).

In spleens and lymph nodes, no difference between the two strains was found for Th1 and Th17 cell activity; however, the proportion of Tregs was reduced in IRAK3^−/−^ spleens and lymph nodes; Th2 cells were reduced in spleens (**Figures 3C, D**). The ratio of Treg:Th17 cells was also significantly reduced in spleens (**Figure 3D**) and nominally lower in LN (**Figure 3C**). These data were consistent with mean fluorescence intensity of FoxP3 staining in FoxP3^+^ cells that was almost threefold higher in WT versus KO lymph nodes (2,914.50 ± 298.9 vs. 1,159.9 ± 114.9, p = 1.6 × 10^−6^) and spleens (3,015.4 ± 332.3 vs. 1,081.9 ± 96.9, p = 7.4 × 10^−7^) (data not shown). As disease progression was accelerated in IRAK3^−/−^ mice, we measured the proportions of T-cell subsets (**Supplementary Figures 4A, B**) and anti-collagen antibody titers in the pre-onset period (**Supplementary Figure 4C**); a similar reduction in Tregs was observed in IRAK3^−/−^ mice, but anti-collagen antibody titers were lower and not dissimilar between strains at this time. In the spleens of naïve IRAK3^−/−^ mice, there were minor increases in CD4^+^ cells co-expressing Tbet, Gata3, or RORγt (**Supplementary Figures 4D, E**). These data indicate significant systemic differences, particularly in regard to T helper subsets, between the strains in the course of experimental arthritis.

### IRAK3^−/−^ joints have higher T cell-associated gene expression

As there were systemic differences between strains, we then compared gene expression localized to the affected joints to determine in finer detail the differences beyond the nominal differences in paw swelling.

Despite IRAK3^−/−^ and WT mouse joints showing relatively minor clinical differences in CIA, a clear difference between the groups was observed when a panel of genes specific for the Th17 and Treg sub-populations was measured (**Figure 3E**, **Supplementary Figure 5**). IRAK3^−/−^ CIA paws expressed higher levels of *Cd3e* and *Cd4*, suggesting increased numbers of CD4^+^ lymphocytes in the paws of IRAK3^−/−^ mice compared with WT mice. Concomitantly, IRAK3^−/−^ paws expressed higher levels of *Stat3* and *Ahr*, two markers associated with the differentiation of Th17 cells, and significantly higher levels of *FoxP3*, *Il2ra*, *Ctla4*, and *Tnfrsf1b*, which are expressed by Tregs; higher levels of *Il12b*, *Ilb*, *Il6*, and *Il17* were also measured, although the difference did not reach statistical significance. *Tnf* and *Tgfb1* gene expression levels were also significantly higher in the IRAK3^−/−^ group.

## Discussion

### IRAK3 expression and disease status

The influence of IRAK3 on the response to therapy has been observed in RA, in which an IRAK3 polymorphism has been shown to be predictive of response to anti-TNF (36). IRAK3 dysregulation is also associated with accelerated and exacerbated asthma (9) and infectious diseases (37), and increased IRAK3 expression in peripheral blood leucocytes is associated with reduced disease activity in RA (17). Animal models using IRAK3 knockout mice have confirmed its role in inhibiting inflammatory responses, but few studies have compared IRAK3 isoforms (6, 38); the disruption of the death domain has been demonstrated to prevent the immunomodulatory activity of IRAK3 (6). We and others have previously demonstrated relatively high levels of inflammatory gene expression (including TNF, which induces IRAK3) in affected joints in arthritis (31). As blocking TNF *in vitro* has been shown to prevent the induction of IRAK3 (2), this study investigated the role of IRAK3 in an animal model of RA, a disease which is amenable to TNF blockade. We began by determining the isoforms of IRAK3 present in mature Mφ exposed to different inflammatory stimuli. As it lacks most of the death domain, the capacity of the alternative isoform to attenuate TLR/IL-1β signaling is likely to be reduced; however other putative actions, such as guanylate cyclase activity, may still be intact (7). We observed that only the longer classical form of IRAK3 was expressed at the protein level.

### IRAK3 and disease activity

A deficit of Tregs or Treg activity is a feature of the failure of tolerance in autoimmune disease in multiple disease models (39–41). Changes to cell composition in spleens and lymph nodes of IRAK3^−/−^ animals, as has been observed previously (22), may have contributed to reduced antibody class switching. While FoxP3 is a phenotype-defining transcription factor for Tregs, the two main sub-types, natural and inducible, were reduced. Most relevant to the current study is the observation that IRAK3 reduces the severity of disease in experimental autoimmune encephalomyelitis (EAE) by Lui B et al. (38); in that study, IRAK3 deficiency accelerated disease progression and increased the ratio of Th17 to Tregs, which we also observed during arthritis. Reduced CD3^+^FoxP3^+^ T cells were also observed in the spleens of IRAK3-deficient mice in a colorectal cancer model (22). An increase of CD4^+^GATA^+^ lymphocytes was measured in the spleens of WT mice, which also had increased plasma levels of IL-5, which is secreted by GATA3^+^ Th2 lymphocytes (42).

A panel of inflammatory genes was assessed for the IRAK3^−/−^ and WT CIA paws. There was a significant difference in the production of pro-inflammatory mediators in the paws of the IRAK3^−/−^ group. In particular, pro-inflammatory cytokine genes such as *Tnf*, *Il12b*, *Il1b*, *Il6*, and *Il17* were all expressed at higher levels in the IRAK3^−/−^ mice, although the difference was not always statistically significant; the expression of *Tgfb1* and *Il10* was also increased. When genes specific for different T-cell subsets were assessed in arthritic paws, an increased expression of both Th17 and Treg markers was observed. IRAK3^−/−^ arthritic paws expressed higher levels of pan-lymphocytic markers *Cd3* and *Cd4*. *Smad3* and *Foxp3*, which are Treg-associated transcription factors, were higher in IRAK3^−/−^ paws as were *Stat3* and *Il23r*, which are more specific for the Th17 subset. Interestingly, *Ctla4* appeared to be expressed in the IRAK3^−/−^ joints at higher levels. CTLA-4 binding to the B7 family members blocks the co-stimulatory signal represented by CD28-B7; this mechanism is used by nTregs to control T-cell activity and as such is suggested to represent a marker for nTregs. Based on these data, we could hypothesize that Th17 and Tregs are increased in the IRAK3^−/−^ paws. The perturbation of T helper subsets in LN and spleen, and increased T cell-associated gene expression in paws, is suggestive of differences in the mobilization and trafficking of T-cell subsets in the absence of IRAK3.

Fewer significant differences were measured in pre-onset lymph nodes and spleens; however, there were a reduced number of FoxP3^+^Helios^+^ cells in IRAK3^−/−^ LN and spleens, and a lower ratio of Tregs to RORγt^+^ lymphocytes in spleens. This indicates a cellular re-distribution of Th sub-populations between different compartments during the pre- and post-onset periods, although an indirect effect of prolonged and exacerbated inflammation on T-cell FoxP3 expression cannot be discounted. The paws as the primary site of the inflammation would contain more pathological cells, such as Th17, and this phenotype may have been stabilized by the higher levels of IL-6 and TGF-β1.

## Conclusions

A limitation of this study is the moderately different expression profile of IRAK3 between humans and mice. While humans tend to express IRAK3 mainly in Mφ-lineage cells, murine expression is somewhat broader and includes other myeloid cells and epithelial cells. Regardless, the association of accelerated development in some forms of human disease and IRAK3 variants is supportive of the immunosuppressive role of IRAK3. Replicating the effect of IRAK3 by therapeutically targeting IRAK4 has been pursued via the development of IRAK4 inhibitors (43) for the treatment of inflammatory disease. In conclusion, we measured dampened and delayed inflammation in a murine model of arthritis in IRAK3-replete mice, as evidenced by reduced IL-1β and higher IL-5 levels in the plasma and more Tregs in draining lymph nodes and spleens; higher immune cell infiltration was suggested by the gene expression profiles in the affected joints of IRAK3^−/−^ mice. This observation is consistent with the known immunomodulatory role of IRAK3 and is similar to the associated changes to disease progression observed in other contexts. The reduced prevalence of Tregs is likely to be a causative factor as part of a wider perturbation in lymphocyte mobilization and phenotypic distribution.

## Data Availability

Sequences in **Supplementary Figure 2** have been deposited in GenBank and have the Accession numbers PV441886 (Ab) and PV441887 (Aq). The raw data supporting the conclusions of this article will be made available by the authors, without undue reservation.

## Supplementary material

Supplementary Table 1 Genotyping primers Primers 1-8 were used for genotyping A^q^ and A^b^ mice. Primers 9 and 10 were used to clone the sequence in **Supplementary Figure 2A**.

Supplementary Table 2 Taqman qPCR assays Taqman qPCR assays and associated genes used for the measurement of gene expression in murine arthritic paws.

Supplementary Figure 1 Quantification of IRAK3 protein expression in human Mφ Relative band intensity for IRAK3 and β-actin was measured using ImageJ and expressed relative to the unstimulated control. Values are mean+SEM, n=2.

Supplementary Figure 2 Determination of I-A^b^ and I-A^q^ alleles by PCR. **(A)** Genomic sequence comparing A^q^ and A^b^ alleles of *H2-Ab1* (NM_207105.3), exon 3 to 5. Yellow highlighting indicates exons. Asterisks indicate identical sequence between alleles. Underlined text indicates primer binding sites for PCRs in **(C)**. **(B)** Phenotyping of A^q^ and A^b^ alleles by flow cytometry by gating on (i) peripheral blood monocytes in erythrocyte-lysed leucocytes from (ii) A^q^ mice, (iii) heterozygous A^qb^ mice or (iv) A^b^ mice. **(C)** Genotyping PCRs for (i, ii) A^q^ mice, (iii, iv) heterozygous A^qb^ mice or (v, vi) A^b^ mice. **(D)** Samples from **(C)** were tested using two primer pairs per reaction. Primer combinations (and PCR product length in bp) for (C(i-vi)): L-R 1&5(561), 1&7(617), 3&5(457), 3&7(513), 2&6(551), 2&8(608), 4&6(454), 4&8(511). Primers for (D): 1, 4, 6 & 7. See **Supplementary Table 1** for primer details. Thermocycler conditions were as follows: 96°C 120 sec, (96°C 30 sec, 63°C 30 sec, 72°C 20 sec) × 35 cycles, 72°C 120 sec. PCR reactions were run on a 1.5% agarose gel alongside Hyperladder 1kb Plus (Bioline, BIO-33068).

Supplementary Figure 3 Generation and genotyping of IRAK3^–/–^H2q mice. Representative genotyping of **(A)** WT and heterogeneous (IRAK3^–/+^) mice and **(B, C)** IRAK3^–/–^ mice bearing H2q, H2b/q and H2b haplotypes.

Supplementary Figure 4 IRAK3 deficiency reduces CD4^+^RORγt^+^ and CD4^+^Foxp3^+^ cells prior to disease onset The percentages of subsets of CD4^+^ lymphocytes, defined by the expression of the transcription factors Tbet, Gata3, FoxP3, Rorγt, and Helios in **(A)** the lymph nodes (LN) and **(B)** spleens (SPL) of pre-onset IRAK3^-/-^ and WT mice (n=5 and 8, respectively). **(C)** Plasma from pre-onset mice was assayed for anti-collagen IgG1 and IgG2a antibodies; values are mean ± SEM n=6-7/group. Percentages of subsets of CD4^+^ lymphocytes in **(D)** LN and **(E)** SPL from unimmunized, naïve mice; * p<0.05, ** p<0.01, **** p<0.0001, Student’s t-test.

Supplementary Figure 5 Inflammatory gene expression is increased in affected paws of IRAK3^-/-^ mice. Gene expression of immune-related genes in the affected paws of mice 10 days post disease onset relative to *Gapdh* using the ΔΔCT method; * p<0.05, ** p<0.01, *** p<0.001, Student’s t-test, n=8/group.
