# Supplementary material for: IRAK3 is upregulated in rheumatoid arthritis synovium and delays the onset of experimental arthritis
Source: Front Immunol. 2025 Apr 30;16:1468341. doi: 10.3389/fimmu.2025.1468341 (PMC12074951; doi:10.3389/fimmu.2025.1468341)
Supplement: Supplementary Table 1 — Genotyping primers Primers 1-8 were used for genotyping Aq and Ab mice. Primers 9 and 10 were used to clone the sequence in Supplementary Figure 2A . [file Table1.pdf]

# Sup. Table 1

Table 1. Primers

| Number | Primer sequence 5’-3’ |
|---|---|
| 1 | CCACATGGGATTTTAGCTGTTATAAC |
| 2 | CCCACATGGGATTTTAGGTGTTATTAT |
| 3 | AGGAAATCATATCTCTCACCTCACAA |
| 4 | AGGAAATCATATCTCTCACCTCATAG |
| 5 | TCCACCATGACTCTGTGGCA |
| 6 | CCACCATGGCTCTGTGGTG |
| 7 | GCTTCTGGAAAAGAGACAGTGAG |
| 8 | CTTCTGGAAAGGAGACAGTGACA |
| 9 | CATGCTGGAGATGACCCCTCATCA |
| 10 | ACCTGCTGGAGGAGGGCCTC |
