# Supplementary material for: IRAK3 is upregulated in rheumatoid arthritis synovium and delays the onset of experimental arthritis
Source: Front Immunol. 2025 Apr 30;16:1468341. doi: 10.3389/fimmu.2025.1468341 (PMC12074951; doi:10.3389/fimmu.2025.1468341)
Supplement: Supplementary Table 2 — Taqman qPCR assays Taqman qPCR assays and associated genes used for the measurement of gene expression in murine arthritic paws. [file Table2.pdf]

Supplementary Table 2

Taqman assay	*Gene*	*Taqman assay*	*Gene*
Mm00607939_s1	*Actb*	Mm00801778_m1	*Ifng*
Mm00431727_g1	*Agtr2*	Mm00833995_m1	*Ikbkb*
Mm00478932_m1	*Ahr*	Mm00496108_m1	*Ikzf2*
Mm00437762_m1	*B2m*	Mm00439616_m1	*Il10*
Mm00432050_m1	*Bax*	Mm00434165_m1	*Il12a*
Mm00477631_m1	*Bcl2*	Mm01288992_m1	*Il12b*
Mm00437783_m1	*Bcl2l1*	Mm00434210_m1	*Il15*
Mm00437858_m1	*C3*	Mm00439619_m1	*Il17*
Mm00839967_g1	*Ccl19*	Mm00434225_m1	*Il18*
Mm00441242_m1	*Ccl2*	Mm00439620_m1	*Il1a*
Mm00441258_m1	*Ccl3*	Mm00434228_m1	*Il1b*
Mm01302428_m1	*Ccl5*	Mm00518984_m1	*Il23a*
Mm99999051_gH	*Ccr2*	Mm00519943_m1	*Il23r*
Mm00438271_m1	*Ccr4*	Mm00434261_m1	*Il2ra*
Mm00432608_m1	*Ccr7*	Mm00446190_m1	*Il6*
Mm00515420_m1	*Cd19*	Mm00434291_m1	*Il7*
Mm03048248_m1	*Cd274*	Mm01321343_m1	*Kmo*
Mm00483137_m1	*Cd28*	Mm00493071_m1	*Lag3*
Mm00519283_m1	*Cd34*	Mm01328172_g1	*Lrp2*
Mm00483146_m1	*Cd38*	Mm00476361_m1	*Nfkb1*
Mm00599683_m1	*Cd3e*	Mm00479807_m1	*Nfkb2*
Mm00442754_m1	*Cd4*	Mm00440485_m1	*Nos2*
Mm00441895_m1	*Cd40*	Mm01285676_m1	*Pdcd1*
Mm00839636_g1	*Cd68*	Mm00478374_m1	*Ptgs2*
Mm00711660_m1	*Cd80*	Mm00448463_m1	*Ptprc*
Mm00444543_m1	*Cd86*	Mm00441278_m1	*Sele*
Mm01182107_g1	*Cd8a*	Mm00441295_m1	*Selp*
Mm00801606_m1	*Col4a5*	Mm00448744_m1	*Ski*
Mm00432688_m1	*Csf1*	Mm00489637_m1	*Smad3*
Mm00438328_m1	*Csf2*	Mm00484741_m1	*Smad7*
Mm00438334_m1	*Csf3*	Mm00782550_s1	*Socs1*
Mm00486849_m1	*Ctla4*	Mm00850544_g1	*Socs2*
Mm00445235_m1	*Cxcl10*	Mm00439518_m1	*Stat1*
Mm00438259_m1	*Cxcr3*	Mm00456961_m1	*Stat3*
Mm01187091_m1	*Ece1*	Mm00448890_m1	*Stat4*
Mm00438656_m1	*Edn1*	Mm01160477_m1	*Stat6*
Mm00433237_m1	*Fas*	Mm00441941_m1	*Tfrc*
Mm01256734_m1	*Fn1*	Mm00441724_m1	*Tgfb1*
Mm00475162_m1	*FoxP3*	Mm00443258_m1	*Tnf*
Mm99999915_g1	*Gapdh*	Mm00437136_m1	*Tnfrsf18*
Mm00446953_m1	*Gusb*	Mm00441883_g1	*Tnfrsf1a*
Mm00442834_m1	*Gzmb*	Mm00441889_m1	*Tnfrsf1b*
Mm00772352_m1	*H2-Ea*	Mm00442039_m1	*Tnfrsf4*
Mm00439221_m1	*H2-Eb1*	Mm00441899_m1	*Tnfrsf9*
Mm00516004_m1	*Hmox1*	Mm00449197_m1	*Vcam1*
Mm00497600_m1	*Icos*	Mm00437304_m1	*Vegfa*
