# Supplementary figures and images for: IRAK3 is upregulated in rheumatoid arthritis synovium and delays the onset of experimental arthritis
Source: Front Immunol. 2025 Apr 30;16:1468341. doi: 10.3389/fimmu.2025.1468341 (PMC12074951; doi:10.3389/fimmu.2025.1468341)

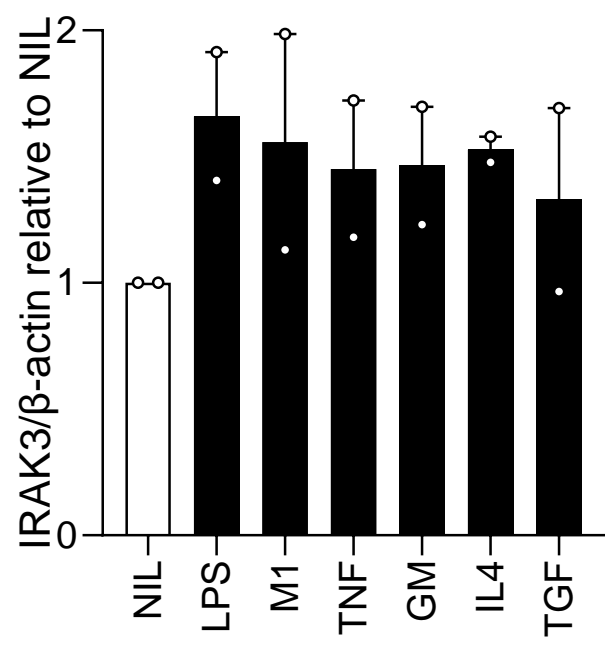

Supplement: Supplementary Figure 1 — Quantification of IRAK3 protein expression in human Mφ Relative band intensity for IRAK3 and β-actin was measured using ImageJ and expressed relative to the unstimulated control. Values are mean+SEM, n=2. [file Image1.pdf]

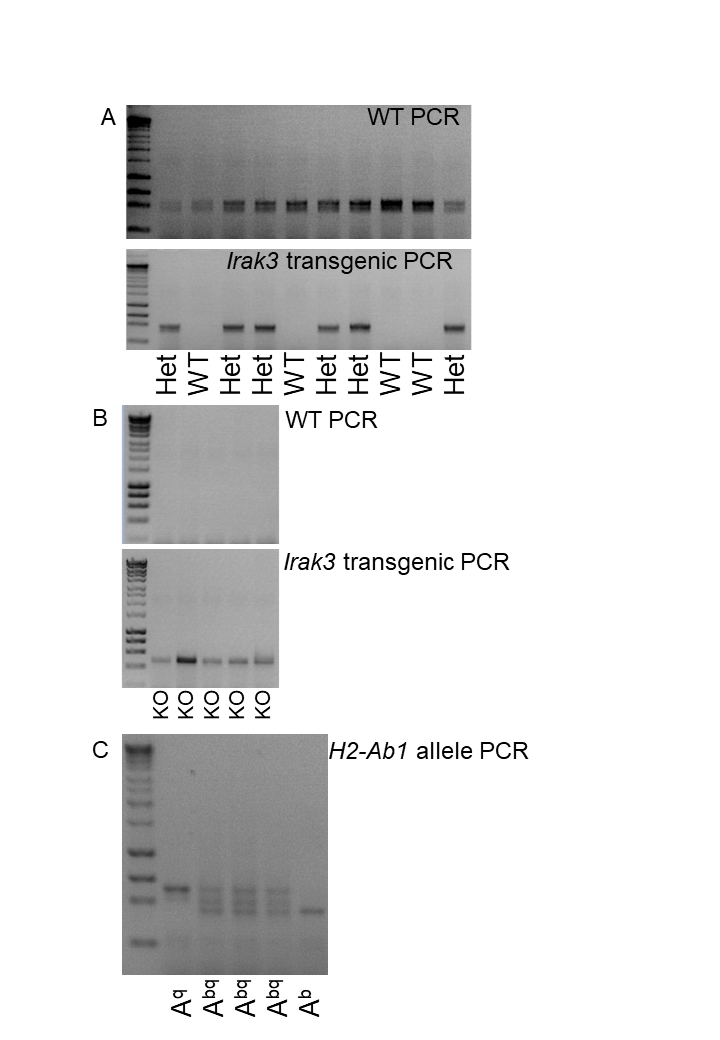

Supplement: Supplementary Figure 3 — Generation and genotyping of IRAK3–/–H2q mice. Representative genotyping of (A) WT and heterogeneous (IRAK3–/+) mice and (B, C) IRAK3–/– mice bearing H2q, H2b/q and H2b haplotypes. [file Image3.tif]

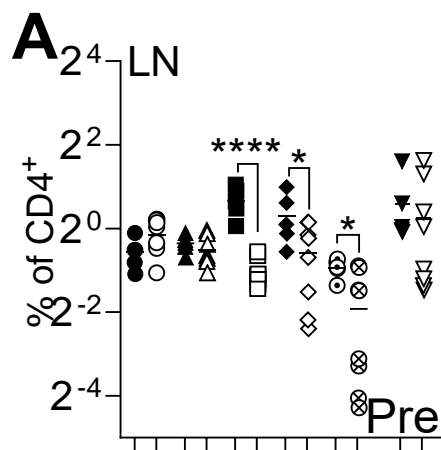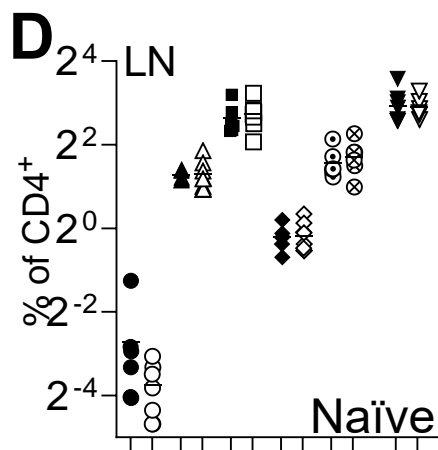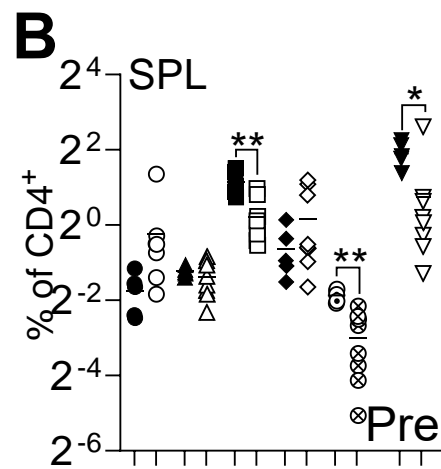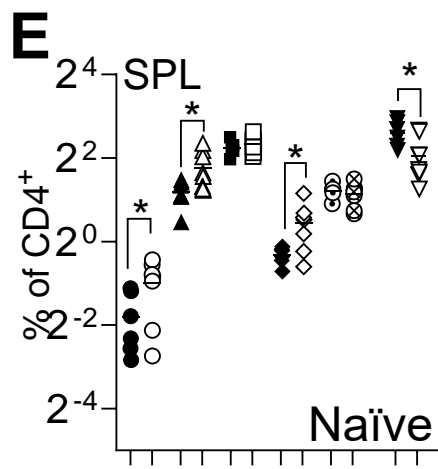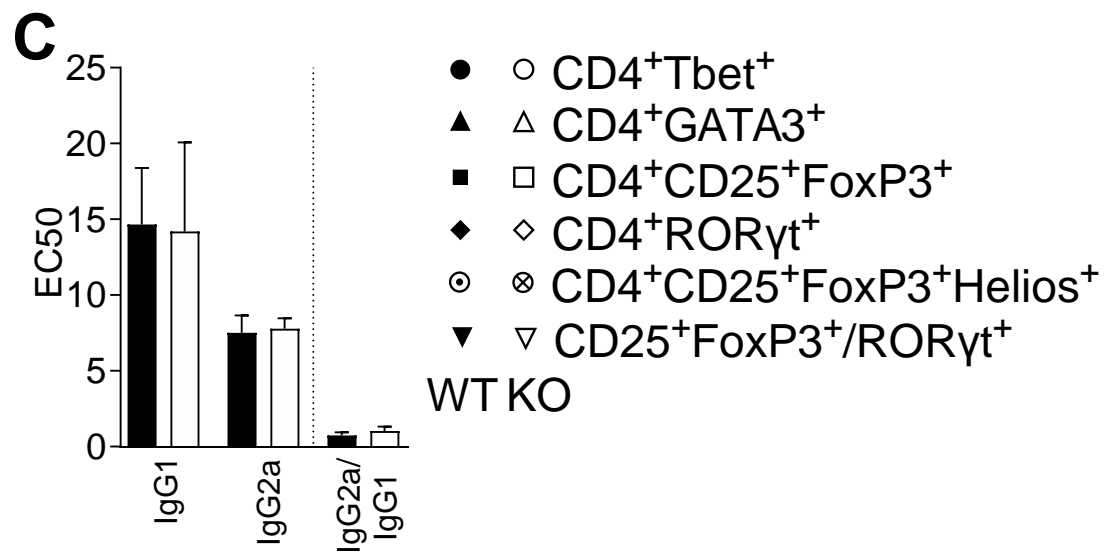

Supplement: Supplementary Figure 4 — IRAK3 deficiency reduces CD4+RORγt+ and CD4+Foxp3+ cells prior to disease onset The percentages of subsets of CD4+ lymphocytes, defined by the expression of the transcription factors Tbet, Gata3, FoxP3, Rorγt, and Helios in (A) the lymph nodes (LN) and (B) spleens (SPL) of pre-onset IRAK3-/- and WT mice (n=5 and 8, respectively). (C) Plasma from pre-onset mice was assayed for anti-collagen IgG1 and IgG2a antibodies; values are mean ± SEM n=6-7/group. Percentages of subsets of CD4+ lymphocytes in (D) LN and (E) SPL from unimmunized, naïve mice; * p<0.05, ** p<0.01, **** p<0.0001, Student’s t-test. [file Image4.pdf]

# Relative Gene Expression

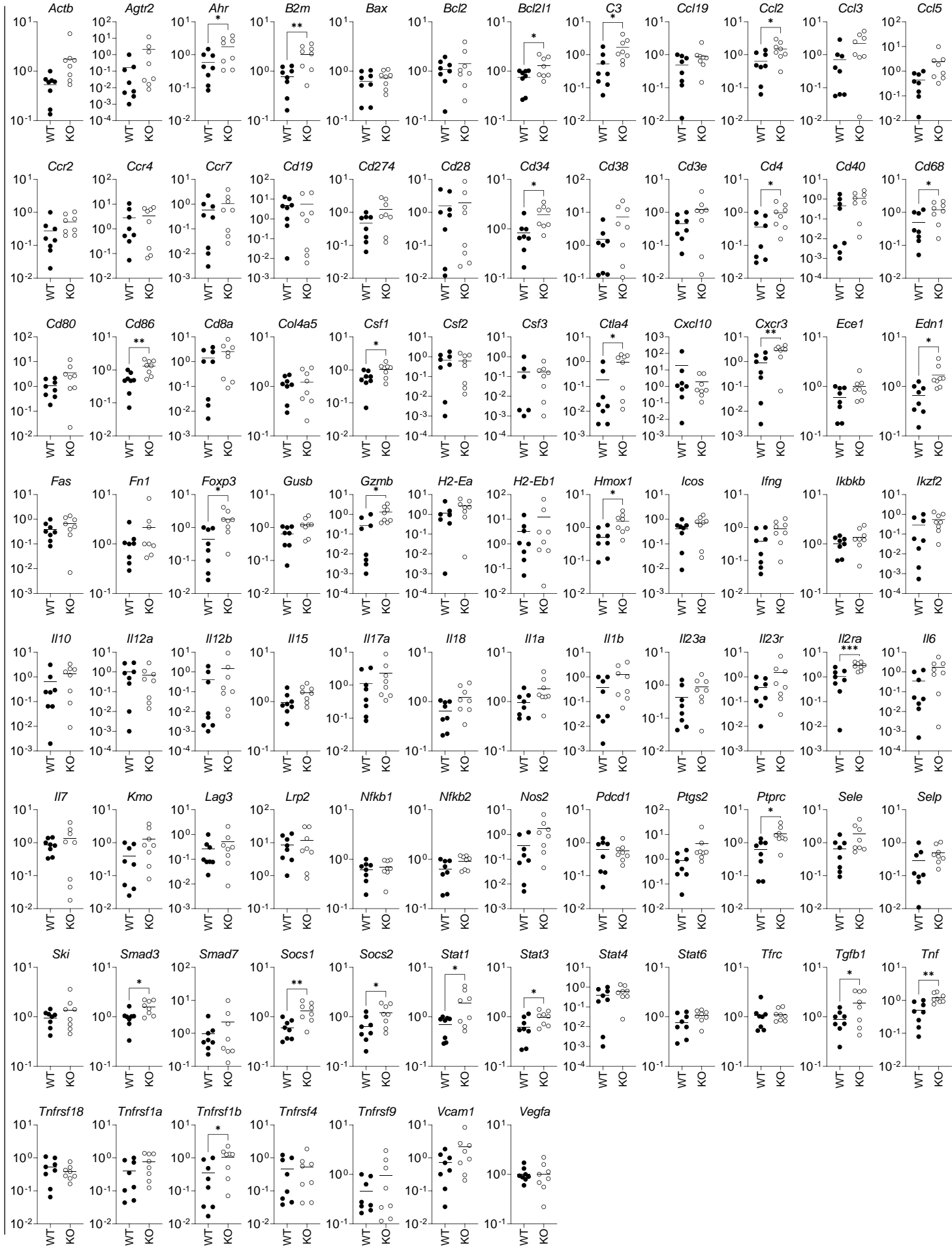

Supplement: Supplementary Figure 5 — Inflammatory gene expression is increased in affected paws of IRAK3-/- mice. Gene expression of immune-related genes in the affected paws of mice 10 days post disease onset relative to Gapdh using the ΔΔCT method; * p<0.05, ** p<0.01, *** p<0.001, Student’s t-test, n=8/group. [file Image5.pdf]
