# Supplementary material for: IRAK3 is upregulated in rheumatoid arthritis synovium and delays the onset of experimental arthritis
Source: Front Immunol. 2025 Apr 30;16:1468341. doi: 10.3389/fimmu.2025.1468341 (PMC12074951; doi:10.3389/fimmu.2025.1468341)
Supplement: Supplementary Figure 2 — Determination of I-Ab and I-Aq alleles by PCR. (A) Genomic sequence comparing Aq and Ab alleles of H2-Ab1 (NM_207105.3), exon 3 to 5. Yellow highlighting indicates exons. Asterisks indicate identical sequence between alleles. Underlined text indicates primer binding sites for PCRs in (C). (B) Phenotyping of Aq and Ab alleles by flow cytometry by gating on (i) peripheral blood monocytes in erythrocyte-lysed leucocytes from (ii) Aq mice, (iii) heterozygous Aqb mice or (iv) Ab mice. (C) Genotyping PCRs for (i, ii) Aq mice, (iii, iv) heterozygous Aqb mice or (v, vi) Ab mice. (D) Samples from (C) were tested using two primer pairs per reaction. Primer combinations (and PCR product length in bp) for (C(i-vi)): L-R 1&5(561), 1&7(617), 3&5(457), 3&7(513), 2&6(551), 2&8(608), 4&6(454), 4&8(511). Primers for (D): 1, 4, 6 & 7. See Supplementary Table 1 for primer details. Thermocycler conditions were as follows: 96°C 120 sec, (96°C 30 sec, 63°C 30 sec, 72°C 20 sec) × 35 cycles, 72°C 120 sec. PCR reactions were run on a 1.5% agarose gel alongside Hyperladder 1kb Plus (Bioline, BIO-33068). [file Image2.pdf]

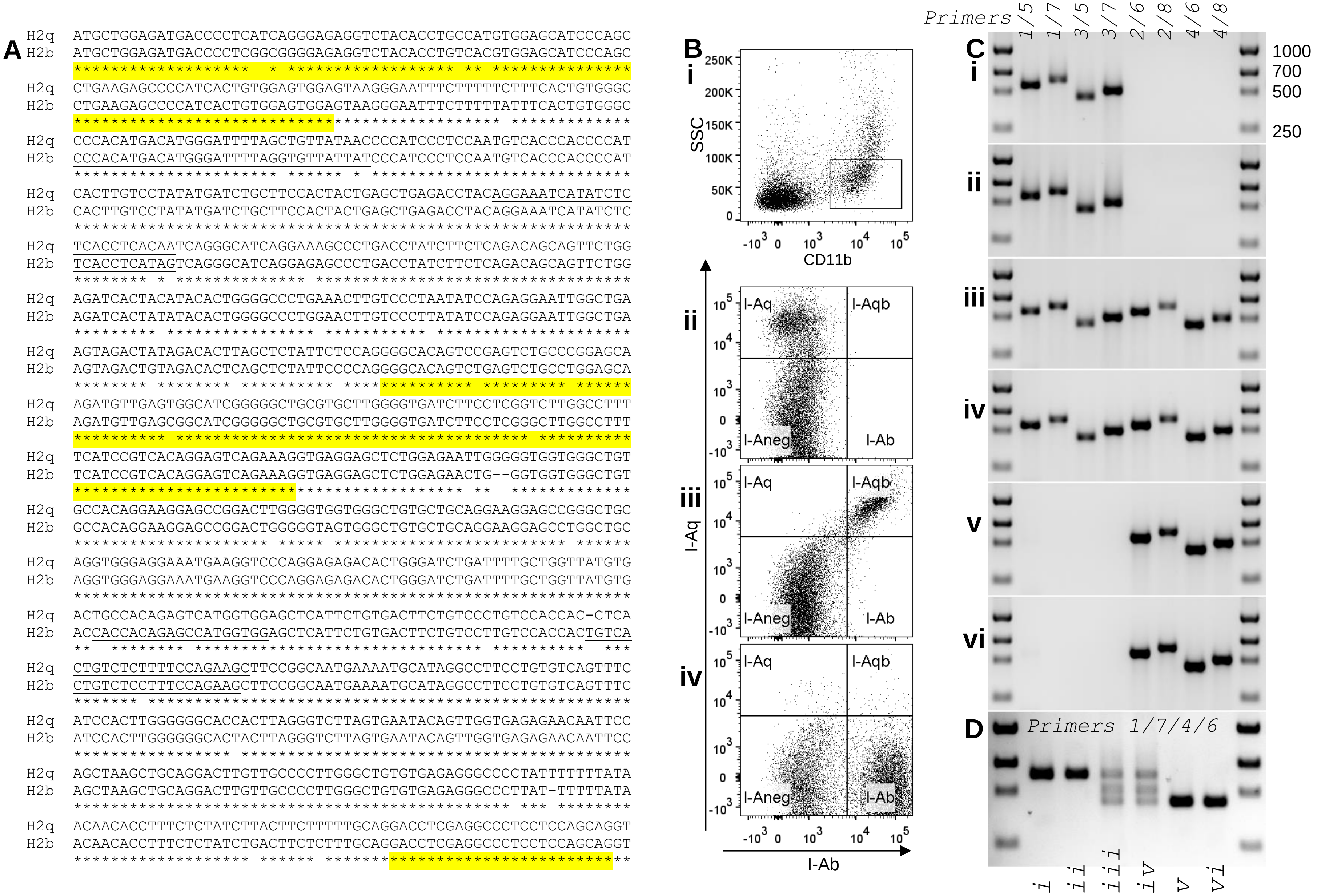
A
H2q ATGCTGGAGATGACCCCTCATCAGGGAGAGGTCTACACCTGCCATGTGGAGCATCCCAGC
H2b ATGCTGGAGATGACCCCTCGGCGGGGAGAGGTCTACACCTGTCACGTGGAGCATCCCAGC
H2q CTGAAGAGCCCCATCACTGTGGAGTGGAGTAAGGGAATTTCTTTTTCTTTCACTGTGGGC
H2b CTGAAGAGCCCCATCACTGTGGAGTGGAGTAAGGGAATTTCTTTTTATTTCACTGTGGGC
H2q CCCACATGACATGGGATTTTAGCTGTTATAACCCCATCCCTCCAATGTCACCCACCCCAT
H2b CCCACATGACATGGGATTTTAGGTGTTATTATCCCATCCCTCCAATGTCACCCACCCCAT
H2q CACTTGTCCTATATGATCTGCTTCCACTACTGAGCTGAGACCTACAGGAAATCATATCTC
H2b CACTTGTCCTATATGATCTGCTTCCACTACTGAGCTGAGACCTACAGGAAATCATATCTC
H2q TCACCTCACAATCAGGGCATCAGGAAAGCCCTGACCTATCTTCTCAGACAGCAGTTCTGG
H2b TCACCTCATAGTCAGGGCATCAGGAGAGCCCTGACCTATCTTCTCAGACAGCAGTTCTGG
H2q AGATCACTACATACACTGGGGCCCTGAAACTTGTCCCTAATATCCAGAGGAATTGGCTGA
H2b AGATCACTATATACACTGGGGCCCTGGAACTTGTCCCTTATATCCAGAGGAATTGGCTGA
H2q AGTAGACTATAGACACTTAGCTCTATTCTCCAGGGGCACAGTCCGAGTCTGCCCGGAGCA
H2b AGTAGACTGTAGACACTCAGCTCTATTCCCCAGGGGCACAGTCTGAGTCTGCCTGGAGCA
H2q AGATGTTGAGTGGCATCGGGGGCTGCGTGCTTGGGGTGATCTTCCTCGGTCTTGGCCTTT
H2b AGATGTTGAGCGGCATCGGGGGCTGCGTGCTTGGGGTGATCTTCCTCGGGCTTGGCCTTT
H2q TCATCCGTCACAGGAGTCAGAAAGGTGAGGAGCTCTGGAGAATTGGGGGTGGTGGGCTGT
H2b TCATCCGTCACAGGAGTCAGAAAGGTGAGGAGCTCTGGAGAACTG--GGTGGTGGGCTGT
H2q GCCACAGGAAGGAGCCGGACTTGGGGTGGTGGGCTGTGCTGCAGGAAGGAGCCGGGCTGC
H2b GCCACAGGAAGGAGCCGGACTGGGGGTAGTGGGCTGTGCTGCAGGAAGGAGCCTGGCTGC
H2q AGGTGGGAGGAAATGAAGGTCCCAGGAGAGACACTGGGATCTGATTTTGCTGGTTATGTG
H2b AGGTGGGAGGAAATGAAGGTCCCAGGAGAGACACTGGGATCTGATTTTGCTGGTTATGTG
H2q ACTGCCACAGAGTCATGGTGGAGCTCATTCTGTGACTTCTGTCCCTGTCCACCAC-CTCA
H2b ACCACCACAGAGCCATGGTGGAGCTCATTCTGTGACTTCTGTCCTTGTCCACCACTGTCA
H2q CTGTCTCTTTTCCAGAAGCTTCCGGCAATGAAAATGCATAGGCCTTCCTGTGTCAGTTTC
H2b CTGTCTCCTTTCCAGAAGCTTCCGGCAATGAAAATGCATAGGCCTTCCTGTGTCAGTTTC
H2q ATCCACTTGGGGGGCACCACTTAGGGTCTTAGTGAATACAGTTGGTGAGAGAACAATTCC
H2b ATCCACTTGGGGGGCACTACTTAGGGTCTTAGTGAATACAGTTGGTGAGAGAACAATTCC
H2q AGCTAAGCTGCAGGACTTGTTGCCCCTTGGGCTGTGTGAGAGGGCCCCTATTTTTTTATA
H2b AGCTAAGCTGCAGGACTTGTTGCCCCTTGGGCTGTGTGAGAGGGCCCTTAT-TTTTTATA
H2q ACAACACCTTTCTCTATCTTACTTCTTTTTGCAGGACCTCGAGGCCCTCCTCCAGCAGGT
H2b ACAACACCTTTCTCTATCTGACTTCTCTTTGCAGGACCTCGAGGCCCTCCTCCAGCAGGT
B
i
SSC
250K
200K
150K
100K
50K
0
-10^3 0 10^3 10^4 10^5
CD11b
ii
iii
iv
I-Aq
I-Aqb
I-Aneg
I-Ab
10^5 10^4 10^3 0 -10^3
I-Ab
Primers
1/5 1/7 3/5 3/7 2/6 2/8 4/6 4/8
C
i
ii
iii
iv
v
vi
1000
700
500
250
D
Primers 1/7/4/6
i ii iii iv v vi
